# Successful Management of Early High-Risk Pulmonary Embolism Following Elective Coronary Artery Bypass Grafting (CABG) Using Intravenous Unfractionated Heparin Alone

**DOI:** 10.7759/cureus.99638

**Published:** 2025-12-19

**Authors:** Christos E Ballas, Christos Tourmousoglou, Polyxeni Oikonomou, Christos Alexiou

**Affiliations:** 1 Department of Cardiothoracic Surgery, University Hospital of Ioannina, Ioannina, GRC

**Keywords:** cabg, cardiac surgery, high-risk pulmonary embolism, thrombocytopenia, unfractionated heparin

## Abstract

Acute pulmonary embolism (APE) following coronary artery bypass grafting (CABG) is uncommon but may rapidly become life-threatening, particularly when it presents in the early postoperative period and is accompanied by hemodynamic instability. We report the case of an 80-year-old woman who developed high-risk APE on the second postoperative day after elective two-vessel CABG. The diagnosis was based on acute hypotension, severe hypoxemia, right ventricular (RV) failure on transthoracic echocardiography, and marked elevation of cardiac biomarkers, while computed tomography pulmonary angiography was not feasible due to profound instability. Management was complicated by recent major surgery and severe postoperative thrombocytopenia, both of which constituted strong contraindications to systemic thrombolysis. Given the prohibitive bleeding risk, the multidisciplinary team initiated continuous intravenous unfractionated heparin (UFH) at the lower therapeutic activated partial thromboplastin time range, combined with vasopressor and inotropic support. Over the following days, the patient demonstrated progressive hemodynamic, respiratory, and renal recovery without bleeding complications. Platelet count gradually normalized, allowing transition to therapeutic low-molecular-weight heparin. She was discharged in stable condition with improved RV systolic function on follow-up echocardiography. This case highlights a rare instance of successful management of early high-risk postoperative APE using UFH alone when reperfusion strategies are contraindicated. The report underscores the critical role of individualized decision-making in the postoperative period and illustrates that, in selected patients, carefully titrated UFH may serve as a lifesaving alternative in situations where thrombolysis or invasive reperfusion therapies cannot be safely performed.

## Introduction

Venous thromboembolic disease (VTD) represents a major cause of morbidity globally, imposing a substantial burden on healthcare systems and affecting an estimated 10 million individuals annually [1]. Despite advances in prevention and diagnostic imaging, acute pulmonary embolism (APE), one of the two principal components of pulmonary VTD, continues to be associated with considerable mortality. When appropriately treated, mortality may be limited to approximately 8%, yet in the presence of hemodynamic instability, it can escalate to nearly 33%. Importantly, up to 10% of patients die suddenly, and more than 60% succumb within the first two hours of presentation. Moreover, APE diagnosed during hospitalization for non-APE indications carries a higher in-hospital mortality than cases identified at initial emergency evaluation [2,3].

Following cardiac surgery, and especially coronary artery bypass grafting (CABG), the diagnosis of APE becomes clinically challenging, as postoperative respiratory symptoms, elevated biomarkers, and right-ventricular (RV) dysfunction may reflect expected post-bypass physiology rather than embolic obstruction. In addition, transfer for confirmatory imaging may be unsafe in unstable patients, thereby complicating management decisions. Although postoperative APE after CABG shows markedly variable incidence across studies, typically remaining relatively uncommon (< 5%), its occurrence is associated with disproportionately high in-hospital mortality [4-7].

Management of APE following cardiac surgery, especially in high-risk cases with hemodynamic instability, which are associated with a mortality of 64% to 74%, represents a major therapeutic challenge. The elevated postoperative bleeding risk commonly contraindicates systemic thrombolysis, forcing individualized reperfusion and anticoagulation strategies amidst increased morbidity and mortality [8].

## Case presentation

We present the case of an 80-year-old woman who was admitted to the cardiothoracic surgery department of a tertiary hospital in Northwestern Greece for scheduled CABG. Her medical history included stable angina, arterial hypertension, and dyslipidemia. Preoperative coronary angiography revealed two-vessel coronary artery disease, consisting of significant proximal left anterior descending artery (LAD) stenosis, significant mid-right coronary artery (RCA) stenosis, and moderate mid-left circumflex artery (LCx) stenosis. The patient underwent two-vessel CABG using the left internal mammary artery to the LAD and a saphenous vein graft to the RCA. Aortic cross-clamp time was 50 minutes, and cardiopulmonary bypass time was 65 minutes. The intraoperative course was uneventful. Postoperatively, she was transferred intubated to the cardiothoracic intensive care unit (ICU), where she remained for 6 hours before successful extubation.

On postoperative day (POD) one, she was mobilized with physiotherapy, tolerated oral feeding, and received prophylactic low-molecular-weight heparin (LMWH) with subcutaneous (SC) bemiparin 3500 international units (IU). She was subsequently transferred to the ward, hemodynamically and respiratory stable. Laboratory tests revealed a platelet count of 105000/µL (preoperative value of 153000/µL), normal renal function, and troponin of 9500 pg/mL in the context of myocardial injury following cardiac surgery. Transthoracic echocardiography (TTE) demonstrated a left ventricular ejection fraction (LVEF) of 50-55% with no segmental wall-motion abnormalities (without changes compared with the preoperative findings), mildly impaired right ventricular (RV) contractility [tricuspid annular plane systolic excursion (TAPSE) 1.45 cm] with normal RV dimensions, as is often observed after cardiac surgery, and no findings suggestive of elevated left ventricular filling pressures or pulmonary hypertension were documented.

**Figure 1:**
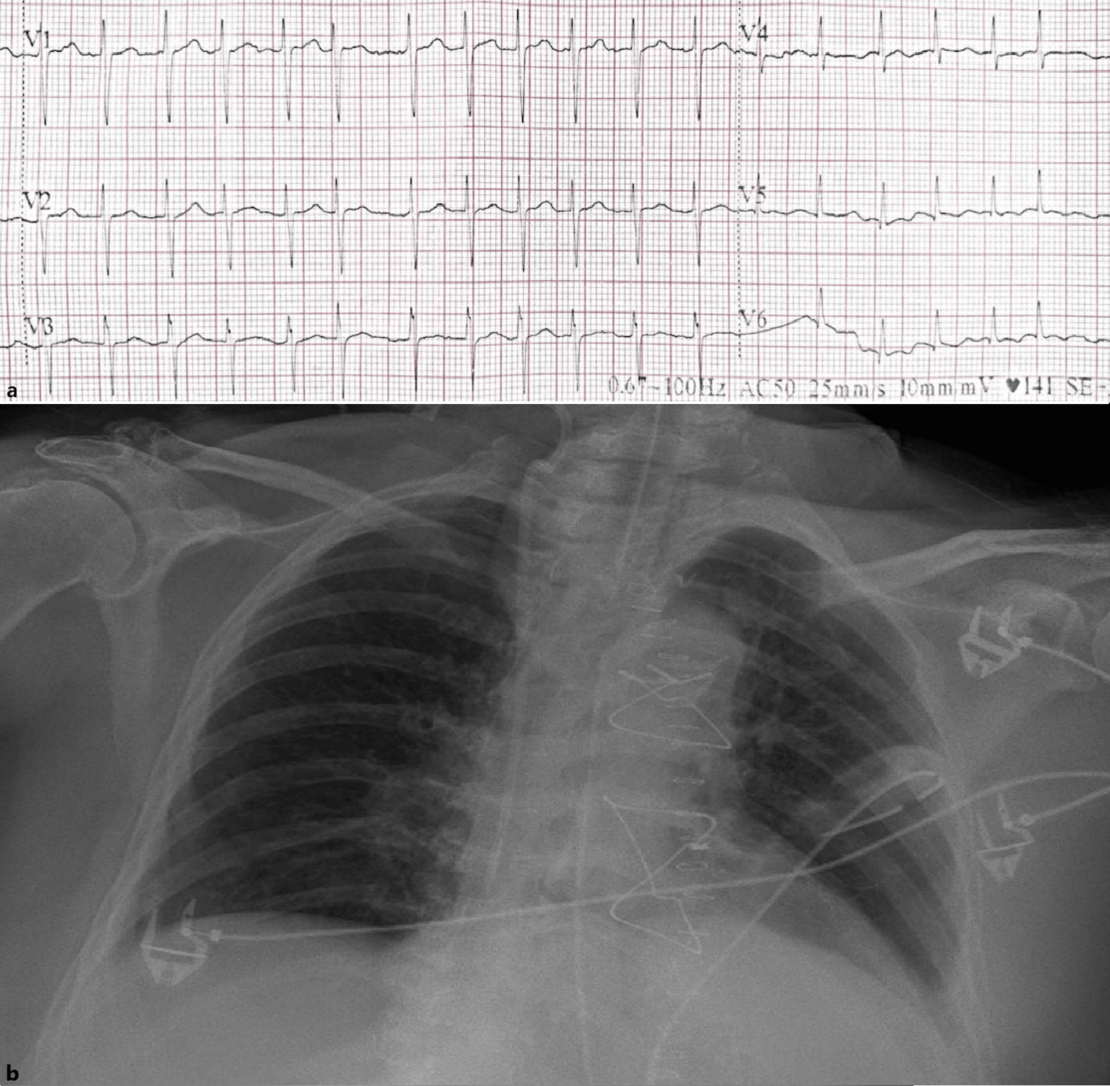
a. A segment of the electrocardiogram demonstrating rapid atrial fibrillation on the second postoperative day. b. Chest radiograph on the second postoperative day.

On POD two, the patient developed sudden-onset dyspnea, tachypnea, impaired consciousness, and hypotension (blood pressure 85/45 mmHg). Chest auscultation revealed decreased breath sounds at both lung bases, while arterial blood gas analysis on 5 L/min oxygen revealed severe hypoxemia (partial pressure of oxygen 53.4 mmHg-oxygen saturation 69.6%) and elevated plasma lactate levels (3.1 mmol/L). Electrocardiography showed rapid atrial fibrillation (AF) at 140 bpm (Figure 1a), whereas the chest X-ray did not demonstrate any definite pathology that could account for the alteration in the patient's clinical status (Figure 1b).

**Figure 2:**
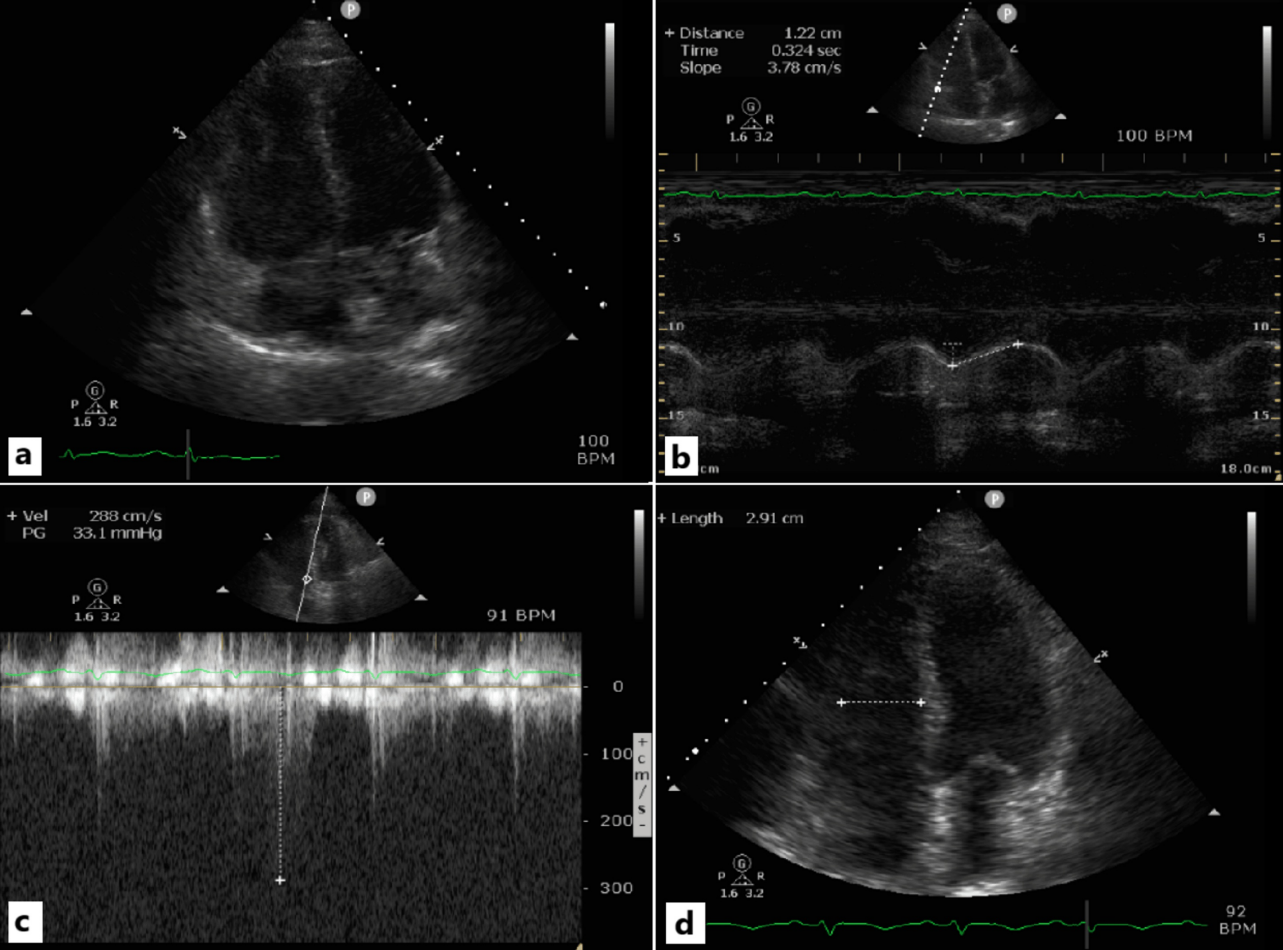
a. Marked enlargement of the RV (apical four-chamber view). Specifically, the ratio of the RV basal diameter to that of the left ventricle is >1. This constitutes a classic echocardiographic marker of RV dysfunction and/or pressure overload in the setting of APE. The measurement was obtained at the time of APE diagnosis. b. Reduced RV contractility based on the TAPSE measurement (apical four-chamber view). The measurement was obtained at the time of APE diagnosis. c. Estimated PASP calculated from the maximal tricuspid regurgitation velocity during the recovery phase (follow-up TTE). PASP is reduced, compared with the estimated value at APE diagnosis, reflecting the effectiveness of therapy in thrombus resolution. d. The estimated mid-cavity diameter of the RV in the apical four-chamber view during the recovery phase is within normal limits. A significant overall reduction in RV dimensions is also evident. RV: Right ventricle/ventricular, APE: Acute pulmonary embolism, PASP: Pulmonary artery systolic pressure, TAPSE: Tricuspid annular plane systolic excursion, TTE: Transthoracic echocardiogram

She was urgently transferred back to the ICU, where vasopressor and inotropic support were initiated alongside oxygen administration via a 40% Venturi mask to maintain oxygen saturation at 90-95%. With norepinephrine administered at 0.1 µg/kg/min, the mean arterial pressure was maintained at approximately 60-65 mmHg. The dose of dobutamine was 7 μg/kg/min. Repeat TTE demonstrated significant deterioration of RV function with RV dilation (increased basal and mid-cavity diameter of the RV at end-diastole in four-chamber view and RV to left ventricular basal diameter ratio at end-diastole >1 in four-chamber view) (Figure 2a) and with an estimated TAPSE of 1.22 cm (Figure 2b), a positive McConnell sign, as well as a dilated inferior vena cava and an increase in PASP to 50-55 mmHg. LVEF remained unchanged, with no new wall-motion abnormalities suggestive of acute postoperative myocardial infarction.

Initially, given the possibility of arrhythmia-induced hemodynamic deterioration, electrical cardioversion of the AF was performed, restoring sinus rhythm, but no change in the hemodynamic profile or improvement in RV contractility was observed. Laboratory tests showed a further decrease in platelet count to 64000/µL, acute kidney injury (urea 68 mg/dL, creatinine 1.33 mg/dL), a marked increase in high-sensitivity troponin (27370 pg/mL), and significant elevation of transaminases, consistent with shock (Table 1). Based on these findings, and relying solely on bedside TTE for imaging, high-risk APE was strongly suspected and considered the most probable diagnosis, despite the fact that the patient was receiving prophylactic LMWH. The probability of APE, based on the revised Geneva score, corresponded to an intermediate clinical likelihood (8 points), while on the PESI scale she scored 210 points, corresponding to a very high 30-day mortality risk.

**Table 1:** Trajectory of the patient’s key laboratory parameters over the inpatient course. PreOP: Preoperative, DS: Day of surgery, POD: Postoperative day, DD: Day of discharge, PLT: Platelet count, Creat: Creatinine, AST: Aspartate transaminase, ALT: Alanine transaminase, HsTN-I: High sensitivity troponin-I

Laboratory Test	PreOP	DS	POD 1	POD 2	POD 3	POD 4	POD 5	DD	Reference Range	Units
PLT	153	138	105	64	26	24	59	156	140-450	10^3^/μl
Urea	37	34	38	68	118	129	122	92	11-54	mg/dl
Creat	0.68	0.84	0.82	1.33	2.27	2.47	2.41	1.33	0.6-1.2	mg/dl
AST	56	82	88	3142	6341	1567	663	37	10-35	IU/l
ALT	12	15	20	2065	3423	1886	1087	110	10-35	IU/l
HsTN-I	28.3	1259.3	9500	27370	32957	14784	4910	64.5	0-11.6	pg/ml

Her hemodynamic condition did not permit transfer for computed tomography pulmonary angiogram (CTPA) imaging. In addition, the continuous decline in platelet count, in combination with recent surgery, excluded systemic thrombolysis due to the markedly elevated hemorrhagic risk. Had there been no contraindication from bleeding risk, thrombolysis could have been performed based solely on bedside TTE findings, without CTPA imaging-if the latter was not feasible (in accordance with European guidelines for APE management [9]). Hemodynamic response to inotropic support was satisfactory, while respiratory function was maintained with high-flow oxygen therapy. Consequently, the “time window” provided by intensive care stabilization allowed consideration of an alternative therapeutic approach, namely unfractionated heparin (UFH) monotherapy, serving as a bridge to decision-making (consideration of surgical or interventional therapy). IV unfractionated heparin (UFH) was initiated with a target activated partial thromboplastin time (aPTT) of 46-70 seconds.

On POD three, blood pressure gradually improved, and vasopressor requirements decreased (halving of the doses of both norepinephrine and dobutamine) with achievement of a mean arterial pressure at 65-70 mmHg. RV function demonstrated improvement, with a significant reduction in chamber dimensions and improvement of TAPSE (1.30 cm). Platelets, however, fell to 26000/µL, necessitating continuation of UFH at the lower therapeutic aPTT range (46-50 seconds). No bleeding complications occurred. Consideration of surgical or interventional therapy began to be abandoned, given the patient’s favorable response to initial treatment.

On POD four, platelets remained low (24000/µL), but respiratory and hepatic parameters showed improvement. Vasopressors were successfully discontinued, and the mean arterial pressure was maintained at 65-70 mmHg. The patient demonstrated adequate urine output, and plasma lactate levels remained within normal limits.

On POD five, platelet recovery (>50000/µL) was documented along with continued clinical stabilization. Renal function parameters stabilized, with consistently adequate hourly urine output (Table 1). Owing to the patient’s clinical and laboratory improvement, no additional diagnostic investigations were pursued. The risk of contrast-induced nephrotoxicity and potential further deterioration of renal function with CTPA imaging was carefully considered. Likewise, no further therapeutic intervention was undertaken. In this context, UFH administration ultimately served as a bridge to recovery in our patients. On the evening of the same day, UFH was discontinued, and administration of LMWH was initiated at a dose adjusted to renal function.

The patient was discharged six days later with substantially improved renal function and no further complications. Follow-up TTE demonstrated recovery of RV function, with the estimated pulmonary artery systolic pressure (PASP) reduced to ~40 mmHg (Figure 2c) and normalization of RV dimensions (mid-cavity diameter = 2.91 cm in the four-chamber view) (Figure 2d).

## Discussion

In suspected high-risk ΑPE, current guidelines permit bedside TTE-based diagnosis when immediate imaging is unfeasible [9]. In our case, we relied exclusively on TTE evidence of acute RV dysfunction and pressure overload to support diagnosis and proceed with urgent management without delay, accepting the risk of potential diagnostic inaccuracy (as confirmation by CTPA was not feasible). This point represents the principal limitation of the present case report.

High-risk pulmonary APE requires immediate reperfusion therapy. Standard management includes systemic thrombolysis, while UFH serves either as initial anticoagulation or as bridging therapy. Candidates for reperfusion therapy may require systemic thrombolysis, catheter-directed intervention, or surgical pulmonary embolectomy, often with extracorporeal membrane oxygenation support in the latter two approaches [9,10].

The management of high-risk APE shortly after cardiac surgery is particularly challenging. In our patient, systemic thrombolysis was avoided because a recent sternotomy constitutes a major contraindication [11]. In addition, thrombocytopenia, which was evident from POD one and progressed to severe thrombocytopenia, further increased the patient’s bleeding risk. Post-CABG thrombocytopenia occurs in over 30% of patients, typically between days two and three, but severe thrombocytopenia is uncommon [12,13]. Furthermore, neither surgical nor percutaneous intervention was initially pursued, given the patient’s compromised condition and the need for a rapid and effective therapeutic decision, thereby avoiding system-related delays.

There are very limited data on the use of thrombolysis after cardiac surgery. Historical and contemporary series report major bleeding rates of > 50% when systemic thrombolysis is given within one week of surgery, decreasing but still significant even if performed later [14]. Regarding UFH, the data supporting its use for APE after cardiac surgery are very limited. Most of the literature addresses prophylactic rather than therapeutic anticoagulation. A systematic review and meta‑analysis found that VTE prophylaxis (UFH or LMWH) after cardiac surgery reduced APE risk (relative risk ≈ 0.45), but studies reported very few cases of established APE being treated with full‑dose heparin [15].

There are some reports of treating high‑risk APE with UFH alone, but these are scarce and generally not encouraging. In a small randomized trial by Jerjes‑Sánchez et al., eight patients with massive APE were assigned to either streptokinase plus heparin or heparin alone: all four patients in the heparin‑only arm died within one to three hours, while those given streptokinase survived [16]. Observational data also suggest that relying solely on anticoagulation in high‑risk APE carries very high mortality: for example, guidelines note that despite anticoagulation, mortality in “massive” APE remains very high. A more recent “target‑trial emulation” involving 1,060 patients with high-risk APE did not include a strategy of heparin alone and demonstrated that advanced reperfusion (systemic thrombolysis, surgical, or catheter‑directed) was associated with substantially lower in‑hospital death than “bridge” strategies without reperfusion [17,18].

In our case, hemodynamic stabilization was achieved through vasopressor and inotropic support combined with low-target continuous IV UFH, without bleeding complications and without the need for systematic thrombolysis, percutaneous, or surgical intervention. Therapy that would normally serve as a bridge to thrombolysis or interventional/surgical reperfusion ultimately functioned as a bridge to recovery.

## Conclusions

The present case highlights the diagnostic and therapeutic complexity of suspected high-risk APE in the immediate post-cardiac surgery setting, particularly when confirmatory imaging is not feasible. Although bedside TTE and clinical deterioration strongly supported a working diagnosis of high-risk APE, diagnostic certainty could not be established. In this context, UFH was selected not as a definitive reperfusion strategy but as a risk-adapted option in view of prohibitive bleeding risk, thrombocytopenia, and recent sternotomy. The observed clinical improvement should be interpreted cautiously and cannot be attributed solely to anticoagulation. Rather, the case underscores the need for individualized decision-making when standard reperfusion pathways are contraindicated, and it reinforces the importance of further evidence guiding management in postoperative patients in whom both diagnosis and treatment remain constrained.

## References

[1] Shah IK, Merfeld JM, Chun J, Tak T (2022). Pathophysiology and management of pulmonary embolism. Int J Angiol.

[2] Bĕlohlávek J, Dytrych V, Linhart A (2013). Pulmonary embolism, part I: Epidemiology, risk factors and risk stratification, pathophysiology, clinical presentation, diagnosis and nonthrombotic pulmonary embolism. Exp Clin Cardiol.

[3] Ballas C, Lakkas L, Kardakari O (2024). In-hospital versus out-of-hospital pulmonary embolism: clinical characteristics, biochemical markers and echocardiographic indices. J Cardiovasc Dev Dis.

[4] Khoury H, Lyons R, Sanaiha Y (2020). Deep venous thrombosis and pulmonary embolism in cardiac surgical patients. Ann Thorac Surg.

[5] Josa M, Siouffi SY, Silverman AB (1993). Pulmonary embolism after cardiac surgery. J Am Coll Cardiol.

[6] Shammas NW (2000). Pulmonary embolus after coronary artery bypass surgery: a review of the literature. Clin Cardiol.

[7] Rao G, Zikria EA, Miller WH (1975). Incidence and prevention of pulmonary embolism after coronary artery surgery. Vasc Surg.

[8] Calé R, Ascenção R, Bulhosa C (2025). In-hospital mortality of high-risk pulmonary embolism: a nationwide population-based cohort study in Portugal from 2010 to 2018. Pulmonology.

[9] Konstantinides SV, Meyer G, Becattini C (2020). 2019 ESC Guidelines for the diagnosis and management of acute pulmonary embolism developed in collaboration with the European Respiratory Society (ERS): The Task Force for the diagnosis and management of acute pulmonary embolism of the European Society of Cardiology (ESC). Eur Heart J.

[10] Weinberg I, Jaff MR (2014). Accelerated thrombolysis for pulmonary embolism: will clinical benefit be ULTIMAtely realized?. Circulation.

[11] Kearon C, Akl EA, Ornelas J (2016). Antithrombotic therapy for VTE disease: CHEST guideline and expert panel report. Chest.

[12] Hamid M, Akhtar MI, Naqvi HI, Ahsan K (2017). Incidence and pattern of Thrombocytopenia in cardiac surgery patients. J Pak Med Assoc.

[13] Griffin BR, Bronsert M, Reece TB (2020). Thrombocytopenia after cardiopulmonary bypass is associated with increased morbidity and mortality. Ann Thorac Surg.

[14] Condliffe R, Elliot CA, Hughes RJ (2014). Management dilemmas in acute pulmonary embolism. Thorax.

[15] Kucher N, Goldhaber SZ (2005). Management of massive pulmonary embolism. Circulation.

[16] Jerjes-Sanchez C, Ramírez-Rivera A, de Lourdes García M (1995). Streptokinase and heparin versus heparin alone in massive pulmonary embolism: a randomized controlled trial. J Thromb Thrombolysis.

[17] Ho KM, Bham E, Pavey W (2015). Incidence of venous thromboembolism and benefits and risks of thromboprophylaxis after cardiac surgery: a systematic review and meta-analysis. J Am Heart Assoc.

[18] Stadlbauer A, Verbelen T, Binzenhöfer L (2025). Management of high-risk acute pulmonary embolism: an emulated target trial analysis. Intensive Care Med.

